# Quality of life assessment after total knee arthroplasty in patients with Parkinson’s disease

**DOI:** 10.1186/s12891-022-05176-1

**Published:** 2022-03-09

**Authors:** Yaqi Zong, Congqiang Hao, Yingjian Zhang, Shuwen Wu

**Affiliations:** Department of Orthopedics, Bao Di Clinical College of Tianjin Medical University, Bao Di Hospital, 8 Guangchuan Road, Baodi District, Tianjin, 301800 Tianjin China

**Keywords:** Total Knee Arthroplasty, Knee Osteoarthritis, Parkinson’s Disease, The Quality of Life

## Abstract

**Background:**

The number of Parkinson’s patients (PD) undergoing total knee arthroplasty (TKA) is increasing. The purpose of the study was to characterize quality of life (QOL) outcomes for patients with coexisting PD and knee osteoarthritis (KOA) following TKA.

**Methods:**

Patients with coexisting PD and KOA undergoing TKA between June 2014 and June 2020 were included. These patients were matched to controls with KOA alone by age, gender, basic social background information and Knee society score (KSS). The primary measure was to assess the QOL by the absolute changes in the EuroQOL5-Dimensions (EQ-5D), Pain and Disability Questionnaire (PDQ), and Patient Health Questionnaire-9(PHQ-9) at the last follow-up (LFU). Secondary measures were changes in QOL that exceeded the minimum clinically important difference value (MCID). Data on the health status and QOL of all patients were collected. Simple and multivariate regression analysis was used to evaluate the impact of PD on their QOL.

**Results:**

Twelve KOA patients with PD were compared with 48 controls. Control patients experienced QOL improvement across all three measures:EQ-5D index (0.545–0.717, *P* < 0.01), PDQ (81.1–52.3, *P* < 0.01) and PHQ-9(8.22–5.91, *P* < 0.01) were significantly improved at the LFU; while in patients with PD, only PDQ (91.0–81.4, *P* = 0.03) slightly improved. There were significant differences in the improvement of QOL between PD patients and the control group through EQ-5D (0.531 vs.0.717, *P* < 0.01) and PDQ (81.4vs.52.3, *P* < 0.01) at the LFU.

**Conclusion:**

TKA has no benefit of QOL beyond a slight improvement in pain-related disability in the KOA patients with PD.

## Background

Parkinson’s disease (PD) is the second most common neurodegenerative disease in the world, with an estimation that 0.3% of the world’s population and 1% of people over the age of 60 have PD [1, 2].The prevalence of PD in nursing homes is more than 5%, and the number of PD patients is expected to continue to increase with the aging of the population [3, 4]. PD patients mainly present with motor symptoms, including tremor, stiffness, unstable or bradykinesia, postural instability, and gait disorders [5–7]. All of these symptoms put PD patients at increased risk for complications after total knee arthroplasty (TKA) [8]. Therefore, Oni and Mackenney [9] have suggested that TKA causes further knee stiffness or flexion contracture in PD patients and so TKA is not recommended for PD patients. However, Vince KG [10] soon demonstrated that TKA in patients with Parkinson’s disease achieved satisfactory surgical results, i.e. reduced knee pain and improved function. Today, as the population ages and life expectancy increase, the prevalence of PD and knee osteoarthritis (KOA) is expected to increase year by year, so it is likely that the number of TKAs will increase, eventually lead to the number of TKAs performed on PD patients gradually increase.

KOA restricts activities such as walking, stair climbing and self-care, affecting people’s quality of life and affecting people not only physically, but also mentally and socially [11]. Surgery is the treatment for end-stage of KOA, which usually has satisfactory results and can significantly improve the quality of life (QOL) [12–17]. Studies have shown that postoperative TKA patients with PD can improve knee function, but the improvement effect is not as good as that of non-PD patients, so PD patients should be given a detailed explanation before receiving TKA [18]. Currently, no studies have assessed the QOL after TKA in patients with KOA and PD. Understanding the impact of surgery on QOL enables appropriate patient management and the prevention of unnecessary surgical interventions.

Therefore, in this study, we evaluated the QOL after TKA in patients coexisting KOA and PD. We hypothesized that all patients would benefit from TKA, but patients with PD would have less benefit in QOL.

## Methods

We retrospectively reviewed all patients with PD and KOA who underwent TKA in our hospital between June 2014 and June 2020.Four non-PD patients with KOA were matched as the control group to 1 KOA patient with PD according to the basic characteristics. Patients who cannot complete preoperative or postoperative QOL self-assessment questionnaires were excluded.

This study was approved by the Ethics Committee of Tianjin Medical University. All methods were in accordance with Declaration of Helsinki, and participants provided written informed consent.

### Data collection

Clinical and demographic data were collected retrospectively from electronic medical records. The orthopedic surgeons and neurologists confirmed the diagnosis of KOA and PD by history-taking, physical examination, and imageological examination. QOL data were collected preoperatively and at the last follow-up (LFU), and all patients were followed up for at least 6 months.

Preoperative and postoperative QOL data were collected prospectively from a patient-reported health status database, including a valid questionnaire at each outpatient clinic. The collected questionnaires included EuroQOL5-Dimensions (EQ-5D) [19–21], Pain and Disability Questionnaire (PDQ) [22, 23], and Patient Health Questionnaire-9 [24–26].Declines in values other than EQ-5D indicate improvements in QOL.

EQ-5D includes five indicators of a patient’s health status: mobility, self-care, usual activities, pain and discomfort, and anxiety and depression. Each indicator is scored 1–3 points, for example, mobility indicators, patients were asked to choose which option they thought appropriate at the moment: (1) “ fully capable of mobility,” (2) “moderate capable of mobility,” or (3) “ not capable of mobility.” The EQ-5D index is derived from each of the five indicators, and ranges from 0 (death) to 1 (excellent health) to represent a patient’s overall health. EQ-5D has been shown to be effective in patients with Parkinson’s disease [27], The Minimal Clinically Important Difference (MCID) of the EQ-5D index was 0.1 [28, 29].

PDQ is often used to evaluate chronic disabling musculoskeletal disorders, focuses on the assessment of pain-induced disability and the ability to perform daily tasks. It includes both functional and psychosocial pain, with the total score of 0–150 points. The MCID of PDQ was 20 [23].

The PHQ-9 evaluates depression based on nine criteria, with each scores ranging from 0 (“ not at all “) to 3 (“ almost every day “), on an overall scale of 0 to 27.The effectiveness of PHQ-9 has been recognized in multicenter analysis [25, 26], and it has also been used as a screening tool for depression in PD patients [30, 31].The MCID of PHQ-9 was 5 [32].

### Data analysis

SPSS 20.0 (SPSS Inc., Chicago, IL, USA) was used to analyze the data. The paired t test and Wilcoxon signed rank test were used to compare the changes of QOL within groups for parametric and non-parametric continuous variables. Fisher’s exact test was used to compare categorical variables between groups. Multivariate regression was used to adjust for confounding covariates and to determine the relationship between PD and postoperative QOL changes. To avoid overfitting, only the variables shown to be related to the outcome variables of the study (through simple regression, *P* < 0.20) were included in the multivariate model, and we applied two sets of models: the first group showed absolute change in EQ-5D/PDQ/PHQ-9 (postoperative minus preoperative value), while the second group showed improvement in EQ-5D/PDQ/PHQ-9 over MCID. Preoperative QOL scores were included in both models to adjust for ceiling effects and to standardize changes in baseline scores.

## Result

A total of 60 patients with TKA were included in this study, including 12 PD patients and 48 non-PD patients (Table 1). The 60 patients were treated by 10 orthopedists, and four of the orthopedists operated on PD patients. The surgical technique was the same in all cases, and bilateral TKA was 25% in PD patients and 29% in the control group.

**Table 1:** Patients’ characteristics

Basic characteristics	PD group	Matched group
Number	12	48
Hoehn-Yahr Stage (mean, SD, range)	2.3, 0.9, 1–4	–
Sex, female	4 (33%)	16 (33%)
Age, year	65.4 ± 11.4	65.2 ± 10.9
Body mass index(BMI)	26.7 ± 4.5	27.7 ± 6.5
Marital status, married	11(92%)	45(94%)
Knee society score(KSS)	35.71 ± 16.84	36.58 ± 15.91
Duration of symptoms (year)	7.6 ± 5.5	8.1 ± 4.7
Bilateral	3 (25%)	14 (29%)
Follow-up time (month)	13.8 ± 7.2	14.5 ± 8.3
Habits and comorbidities		
Smoking	3 (25%)	13 (27%)
Alcoholism	0 (0%)	2 (4%)
Hypertension	5 (42%)	19 (40%)
Hyperlipidemia	3 (25%)	14 (29%)
Diabetes	2 (17%)	11 (23%)
Depression/anxiety	3 (25%)	10 (21%)
Cardiac failure	0 (0%)	1 (2%)
Coronary heart disease	2 (17%)	7 (15%)
Osteoporosis	3 (25%)	13 (27%)
History of cancer	1 (8%)	3 (6%)
History of falls	4 (33%)	14 (29%)

*Note*: Continuous variables are expressed as mean ± standard deviation; Classification variables are expressed as quantities (%)

All patients completed the QOL questionnaires. PD patients were followed up for a mean of 13.8 months after surgery, while the control group was followed up for a mean of 14.5 months (*P* = 0.79). There were no significant differences in the basic characteristics of the patients, and the underlying disease, preoperative symptom duration, KSS, surgical method, and postoperative follow-up time were the same.

### QOL outcomes

QOL assessed by these three questionnaires in patients with preoperative KOA was similar regardless of whether PD was associated (Table 2). There was no significant difference in EQ-5D scores among all groups. For example, there was no statistically significant difference in EQ-5D index between PD group (0.559) and control group (0.545, *P* = 0.81) before surgery (*P*>0.05). Similarly, there was no significant difference in QOL assessed by total PDQ between the PD group (91.0) and the control group (81.1, *P* = 0.18). The preoperative PHQ-9 of PD patients was 9.48, and that of control group was 8.22, *P* = 0.49, and the difference was not statistically significant.

**Table 2:** Unadjusted QOL outcomes

Measurement index	PD group (*N* = 12)	*P*^1^	Matched group (*N* = 48)	*P*^1^	*P*^2^
EQ- mobility					
Pre-operative	1.89 ± 0.30		1.94 ± 0.34		0.79
Post-operative	1.89 ± 0.29	1.00	1.78 ± 0.39	0.03	0.29
EQ- self-care					
Pre-operative	1.72 ± 0.47		1.49 ± 0.58		0.18
Post-operative	1.72 ± 0.55	1.00	1.35 ± 0.49	0.03	0.05
EQ-usual activities					
Pre-operative	2.09 ± 0.54		2.09 ± 0.47		1.00
Post-operative	2.27 ± 0.65	0.63	1.79 ± 0.56	< 0.01*	0.02*
EQ- pain and discomfort					
Pre-operative	2.19 ± 0.60		2.40 ± 0.57		0.31
Post-operative	2.37 ± 0.49	0.62	1.92 ± 0.58	< 0.01*	0.03
EQ- anxiety/depression					
Pre-operative	1.72 ± 0.64		1.58 ± 0.55		0.55
Post-operative	1.46 ± 0.51	0.26	1.31 ± 0.55	< 0.01*	0.33
EQ-5D index					
Pre-operative	0.559 ± 0.220		0.545 ± 0.218		0.81
Post-operative	0.531 ± 0.202	0.48	0.717 ± 0.208	< 0.01*	0.01*
PDQ- function					
Pre-operative	57.8 ± 26.3		46.7 ± 21.6		0.18
Post-operative	53.3 ± 21.0	0.04	32.8 ± 24.3	< 0.01*	0.02*
PDQ- psychosocial					
Pre-operative	32.1 ± 18.3		26.0 ± 14.5		0.41
Post-operative	27.3 ± 13.2	0.02*	18.2 ± 16.0	< 0.01*	0.09
PDQ-total					
Pre-operative	91.0 ± 39.4		81.1 ± 33.1		0.18
Post-operative	81.4 ± 34.1	0.03	52.3 ± 35.4	< 0.01*	0.01*
PHQ-9					
Pre-operative	9.48 ± 6.41		8.22 ± 7.02		0.49
Post-operative	8.22 ± 5.16	0.31	5.91 ± 6.45	< 0.01*	0.30
MCID					
EQ-5D	3.25%		27.56%		0.04
PDQ	2.17%		23.48%		0.01*
PHQ-9	1.8%		13.27%		0.01*

*Statistically significant: *P*<0.02

^*1*^Paired t test and Wilcoxon signed rank test are used for intra-group comparisons of parametric and nonparametric continuous variables, respectively

^*2*^The t-test and Fisher’s exact test were used for inter-group comparisons of continuous and categorical variables, respectively

In the matched group (Table 2), except for mobility and self-care (*P* = 0.03), all other EQ-5d indexes were significantly improved at the last follow-up (*P* < 0.01), so the postoperative EQ-5d index was significantly improved (0.545–0.717, *P* < 0.01). In the matched group, PDQ (81.1–52.3, *P* < 0.01) and PHQ-9(8.22–5.91, *P* < 0.01) were also significantly improved at the last follow-up. In contrast, PD patients had only a slight improvement in PDQ (91.0–81.4, *P* = 0.03), but there was no significant difference in EQ-5D (0.559–0.531, *P* = 0.48) or PHQ-9(9.48–8.22, *P* = 0.31) after surgery.

Although there was no significant difference in the QOL between the groups preoperatively, the improvement in QOL of PD patients at final follow-up was significantly worse than that of the matched group, as measured by EQ-5D (0.531 vs.0.717, *P* < 0.01) and PDQ (81.4vs.52.3, *P* < 0.01), with a significant difference.

### Multiple regression analysis of QOL improvements

Multivariate regression analysis was used to adjust for confounding factors between the two groups, and absolute changes in QOL were taken as results (Table 3). Preoperative EQ-5D and PD were important independent predictors of EQ-5D changes. After adjusting for confounding demographic, comorbid, and unilateral or bilateral TKA, PD predicted a decrease in EQ-5D improvement at the LFU (*β* = − 0.09, *P*<0.01).

**Table 3:** Multiple regression model for improvement of QOL (*N* = 60)

Outcome	Variable	βcoefficient	*P*
ΔEQ-5D	Preoperative EQ-5D	−0.48	< 0.01*
	Follow-up (month)	0.00	0.57
	PD	−0.09	< 0.01*
	Married	0.04	0.11
	History of cancer	−0.07	0.09
	Bilateral TKA	−0.03	0.10
ΔPDQ	Preoperative PDQ	−0.38	< 0.01*
	Follow-up (month)	0.31	0.27
	Married	−6.98	0.05
	History of cancer	8.12	0.13
	Bilateral TKA	5.21	0.01*
ΔPHQ-9	Preoperative PHQ-9	−0.39	< 0.01*
	Follow-up (month)	−0.03	0.23
	Married	−0.70	0.15
	Prior depression/anxiety	2.89	< 0.01*
	Duration of symptoms (months)	0.09	< 0.01*

* Statistically significant: *P*<0.02

Outcome: the increase of EQ-5D indicated the improvement of QOL; declines in PDQ and PHQ-9 indicate improvement of QOL

The data confirmed that PD was not an important independent predictor of changes in PDQ, while preoperative PDQ and unilateral or bilateral TKA were proved to be important independent predictors of changes in PDQ. Bilateral TKA (*β* = 5.21, *P* = 0.01) indicated a decrease in postoperative PDQ improvement. Similarly, PD was not a significant independent predictor of changes in PHQ-9. Multivariate regression analysis found that prior depression and anxiety (*β* = 2.89, *P* < 0.01) and longer preoperative duration of symptoms (*β* = 0.09, *P* < 0.01) were predictors of diminished improvement in PHQ-9.

### Multivariate regression analysis with MCID

Multivariate regression analysis was used to adjust for confounders between the two groups, and improvement in QOL over MCID was taken as the result (Table 4). After adjustment for confounders, PD patients failed to achieve the MCID for EQ-5D (OR:0.07, 95%[CI]: < 0.01–0.49, *P* < 0.01). PD was also not a significant independent predictor of PDQ improvement over MCID. The improvement of PHQ-9 in PD patients also did not exceed that in MCID. However, the surgery age (OR:0.91,95%[CI]:0.79–0.97, *P* = 0.02), prior depression and anxiety (OR:0.01, 95%[CI]: 0.01–0.11, *P* < .01), and the duration of symptoms before operation (OR:0.89,95%[CI]:0.84–0.97, *P* < 0.01) was significantly correlated with PHQ-9 failing to reach MCID. In contrast, increased follow-up time was associated with an improvement in PHQ-9 over MCID (OR:1.12, 95%[CI]:1.01–1.27, *P* = 0.02).

**Table 4:** Multivariate regression model with MCID (N = 60)

outcome	variable	OR [95% [CI]]	*P*
ΔEQ-5D > MCID	Preoperative EQ-5D	< 0.01 [< 0.01–0.02]	< 0.01*
	Follow-up (month)	0.97 [0.91–1.03]	0.48
	PD	0.07 [< 0.01–0.49]	< 0.01*
	smoking	0.16 [0.02–1.15]	0.06
ΔPDQ>MCID	Preoperative PDQ	1.01 [0.98–1.04]	0.11
	Follow-up (month)	1.04 [0.97–1.07]	0.69
	Married	2.96 [0.71–14.56]	0.14
	History of cancer	< 0.01 [< 0.01–0.79]	0.03
	Duration of symptoms (months)	0.99 [0.92–1.02]	0.14
ΔPHQ-9 > MCID	Preoperative PHQ-9	1.47 [1.18–2.06]	< 0.01*
	Follow-up (month)	1.12 [1.01–1.27]	0.02*
	Surgery age	0.91 [0.79–0.97]	0.02*
	Prior depression/anxiety	< 0.01 [< 0.01–0.11]	< 0.01*
	Duration of symptoms (months)	0.89 [0.84–0.97]	< 0.01*

*OR* Odds ratio, *CI* Confidence interval, *MCID* Minimal clinically important difference

* Statistically significant: *P*<0.02

## Discussion

Parkinson’s disease is one of the most common neurological disorders in a medical era of increasing life expectancy and an aging population, and TKA is one of the most common procedures for older people. TKA is a safe and effective surgical procedure that can relieve pain and improve the function of the affected limb. The improvement of QOL in the patient of KOA after TKA has been extensively and positively described in the literature [12–17]. However, there is no such study been conducted in KOA patients with PD. We conducted a controlled study to demonstrate that TKA can improve QOL in these particular patients. Our results demonstrated that EQ-5D, PDQ, and PHQ-9 were significantly improved in the control group, while PD patients only showed slightly improvement in QOL assessed by PDQ. Although preoperative QOL were similar, PD patients had significantly worse QOL on all three measures at LFU than the control group.

### QOL in PD patients

Studies have described the effect of symptoms of PD on QOL. Kuopio et al. [33] used the 36-item Health Survey Summary Scale (SF-36) in a community-based sample to investigate the relationship between non-motor symptoms of PD and QOL, and concluded that depression was most closely related to QOL. Barone et al. [34] subsequently conducted a large multicentric study to investigate the incidence of non-motor symptoms and their impact on QOL in patients with PD. Based on 39-item Parkinson’s Disease Questionnaire (PDQ-39), apathy, fatigue, attention, memory and mental symptoms are all detrimental to QOL. Motor symptoms have also been shown to be detrimental to QOL in people with PD. Floden et al. [35] suggested that non-motor symptoms (such as depression) prior to DBS treatment of PD significantly affected the QOL outcomes measured by PDQ-39. There was no correlation between preoperative motor symptoms and QOL as measured by the Unified Parkinson’s disease Rating Scale, Part III. Finally, Soh et al. [36] conducted a systematic review of health-related QOL in PD patients and determined that depression, disease severity, and disability were the most detrimental to the improvement of QOL.

### QOL in PD patients after TKA

TKA has been recognized to improve QOL in patients with KOA. TKA can relieve pain and make patients feel satisfied, and even improve the quality of life of octogenarian patients to the level of septuagenarian patients [37]. Szmyd J et al. [38] also proved that TKA significantly reduced the pain intensity of patients and significantly improved the patient’s ability to live daily. In a 2-year follow-up after TKA, DT Wei et al. [39] found that improvements in knee specific outcomes (KSS and OKS) were closely associated with improvements in health-related quality of life (SF-36). Although the number of PD patients with TKA is relatively small, favorable surgical outcomes with TKA in these patients have occasionally been reported. For example, Sun QC et al. [40] used KSS score to illustrate that for PD patients, both knee function and range of motion were greatly improved after TKA, especially in pain. However, no studies have reported the improvement of QOL in PD patients after TKA. Montiel et al. [41]. questioned the improvement of QOL in PD patients after TKA, but did not draw a conclusion on whether QOL was improved or not. Therefore, we tried to further explain the improvement of QOL in PD patients with TKA.

In this study, the preoperative QOL of PD patients was similar to that of non-PD patients, and there were no significant differences between EQ-5D, PDQ, and PHQ-9 (Table 2). Postoperatively, the control group showed significant improvement in all indicators, while the PD patients only showed slight improvement in PDQ. In addition, compared with the control group, postoperative QOL was significantly worse in PD patients as assessed by EQ-5D (0.531 vs.0.717, *P* < 0.01) and PDQ (81.4vs.52.3, *P* < 0.01), with only a small number of PD patients achieving EQ-5D MCID (25%vs.56%, *P* = 0.04). These results indicate that patients with PD have a poorer improvement in QOL after TKA compared to patients with KOA alone, but still relieve symptoms or pain related disability. The results are not entirely consistent with the view that TKA can significantly improve pain in PD patients [40, 41].More importantly, all of these results indicate that there was no significant improvement in overall QOL after TKA in the PD patient. The reason why the QOL of PD patients cannot be significantly improved after TKA is not clear. It is possible that the disease burden associated with Parkinson’s disease limits improvement in QOL, even if KOA symptoms are alleviated. And another explanation may be that postoperative physical therapy is ineffective in PD patients. The difficulty of diagnosing both PD and KOA at the same time also needs to be considered, as the clinician may not be able to distinguish the degree of influence of KOA and PD on gait changes from symptoms. Therefore, it is possible that the specific gait changes and postural stability of PD in patients make TKA treatment less effective than expected.

Multivariate regression was used to analyze the confounding factors between the control groups. The independent impact of PD on absolute changes in QOL and the improvement in QOL exceeding the MCID (Tables 3, 4). In both models, after adjustment for demographics, comorbidities, surgical characteristics, preoperative QOL, and follow-up time, PD significantly and independently predicted a decrease in EQ-5D improvement (β = − 0.09, *P* < 0.01), and failed to reach the EQ-5D MCID (OR:0.07, 95%[CI]: < 0.01–0.49, *P* < 0.01). Moreover, PD was not associated with improvement in either PDQ or PHQ-9.

QOL measurements are a more global assessment of health. While a poorer surgical outcome may be associated with a poorer QOL, patient satisfaction may relate to relief of pain and improved function of the operated knee. In fact, when asked, most patients prioritize relief of pain. The finding that there is in statistically significant difference in PDQ between the two groups is then especially important. Furthermore, it is not surprising either that patients with PD report a poorer overall QOL as they still are affected by their PD.

## Limitations

As a retrospective study, the data collected by clinicians were not standardized, which led to measurement bias in our results. KOA is a common disease, so it is possible that a small proportion of PD patients may be misdiagnosed as KOA. Preoperative functional and QOL indicators were similar in both groups, however PD did affect limb function and QOL. If the influence of PD on these indicators was removed, the effect of KOA on these indicators would be lower in the PD group. Therefore, it is likely that the degree of severity of knee arthritis in the PD patients undergoing surgery was lower than that in the control group. In addition, the study involved 10 surgeons (4 of whom were involved in the operation of PD patients) who made surgical decisions. Although the procedure was the same, the timing of the surgery might be different, so conservative or aggressive surgeons would choose to perform the surgery on PD patients with severe or mild KOA. These limitations may prevent our results from being generalizable.

PD and KOA may be diagnosed at different stages of their respective disease course, introducing lead-time bias. PD is a progressive disease, so PD patients may not have achieved an overall improvement in QOL at the LFU. We attempted to control for confounders by implementing a matching cohort study design to eliminate differences between cohorts. Due to the small number of KOA patients combined with PD, the sample size of the study is relatively small, which inevitably limits the applicability and accuracy of multivariate regression, as well as the discovery of statistically significant changes in QOL.

Although the limitations of the study design may obscure the benefits of TKA in improving QOL, there is evidence of poor improvement in QOL in PD patients after TKA. Therefore, more prospective, randomized studies are needed to determine the merits of TKA or conservative treatment in KOA patients with PD.

## Conclusion

This is the first study to evaluate the QOL after TKA in KOA patients with PD. Compared with the control group with KOA alone, patients with PD had only a slight improvement in PDQ and had poorer overall QOL improvement. After adjusting for confounding factors, PD predicted a weakening of overall QOL improvement as assessed by EQ-5D and was not associated with improvement in QOL as assessed by either PDQ or PHQ-9. Therefore, data from this study suggest that TKA has no clinical benefit in improving QOL beyond a slight improvement in pain-related disability in the PD population.

## Data Availability

The datasets used and/or analysed during the current study are available from the corresponding author on reasonable request.

## Abbreviations

PD: Parkinson’s patients
TKA: Total knee arthroplasty
QOL: Quality of life
KOA: Knee osteoarthritis
KSS: Knee society score
EQ-5D: EuroQOL5-Dimensions
PDQ: Pain and Disability Questionnaire
PHQ-9: Patient Health Questionnaire-9
LFU: last follow-up
MCID: Minimum clinically important difference value
